# Supplementary material for: End Sequence Analysis Toolkit (ESAT) expands the extractable information from single-cell RNA-seq data
Source: Genome Res. 2016 Oct;26(10):1397–410. doi: 10.1101/gr.207902.116 (PMC5052061; doi:10.1101/gr.207902.116)
Supplement: Supplemental Material [file supp_gr.207902.116_Supp_Materials.tar.gz › ESAT-esat_v0.1/doc/html/classbroad_1_1pda_1_1seq_1_1rap_1_1_slide_and_calculate.html]

ESAT: broad.pda.seq.rap.SlideAndCalculate Class Reference


ESAT


- Main Page
- Related Pages
- Packages
- Classes
- Files

- Class List
- Class Index
- Class Hierarchy
- Class Members

All Classes Namespaces Files Functions Variables Enumerator Pages

- broad
- pda
- seq
- rap
- SlideAndCalculate

Public Member Functions |
Static Public Member Functions |
Public Attributes |
Protected Member Functions |
List of all members

broad.pda.seq.rap.SlideAndCalculate Class Reference

Inheritance diagram for broad.pda.seq.rap.SlideAndCalculate:

[legend]

Collaboration diagram for broad.pda.seq.rap.SlideAndCalculate:

[legend]

|  |  |
| --- | --- |
| Public Member Functions | |
| Iterator<?extends WindowScore > | getWindowScoreIterator () throws IOException |
|  | |
| Public Member Functions inherited from broad.pda.seq.rap.GenomeScoringProgram | |
| WindowProcessor<?extends   WindowScore > | getWindowProcessor () |
|  | |
| WindowProcessor<?extends   WindowScore > | getWindowProcessor (AnnotationCollection<?extends Annotation > target, AnnotationCollection<?extends Annotation > control) |
|  | |
| Public Member Functions inherited from broad.pda.seq.rap.GenomeCommandLineProgram | |
| List< Annotation > | getRegions () |
|  | |
| Map< String, Annotation > | getRegionsMap () |
|  | |
| AnnotationList< Annotation > | getRegionSet () |
|  | |
| Annotation | getGenes () |
|  | |
| Boolean | isGenomicSpace () |
|  | |
| AlignmentModel | loadAlignmentModel (File bamFile) |
|  | |
| AlignmentModel | loadAlignmentModel (File bamFile, boolean pairedEnd) |
|  | |

|  |  |
| --- | --- |
| Static Public Member Functions | |
| static void | main (final String[] args) |
|  | |

|  |  |
| --- | --- |
| Public Attributes | |
| String | USAGE = "Slides across the genome and counts reads (or calculates ratios for two models). This is currently written for Genomic Space analyses but should be modified to allow other coordinate systems." |
|  | |
| int | WINDOW |
|  | |
| int | OVERLAP |
|  | |
| File | OUTPUT |
|  | |
| Double | RPKM\_RATIO\_OFFSET = RatioScore.RPKM\_OFFSET |
|  | |
| Public Attributes inherited from broad.pda.seq.rap.GenomeScoringProgram | |
| File | TARGET |
|  | |
| File | CONTROL = null |
|  | |
| String | SCORE = "count" |
|  | |
| boolean | PAIRED\_END =true |
|  | |
| Public Attributes inherited from broad.pda.seq.rap.GenomeCommandLineProgram | |
| String | SIZES = "/seq/lincRNA/data/mm9/sizes" |
|  | |
| String | MASK\_FILE = "/seq/mguttman/ChIPData/MaskFiles/MM9Segments/all.mask.mouse.n36.d2.bin" |
|  | |
| double | PCT\_MASKED\_ALLOWED = 50.0 |
|  | |
| String | REGION = null |
|  | |
| String | GENE = null |
|  | |
| Integer | MAX\_FRAGMENT\_LENGTH = 10000 |
|  | |
| Integer | MIN\_MAPPING\_QUALITY = 30 |
|  | |
| String | COORD\_SPACE = "genomic" |
|  | |
| String | ANNOTATION = "/seq/lincRNA/Shari/Annotations/RefSeq\_LincV3\_ChromatinWithNames\_nonrandom\_collapsed\_overlappers.bed" |
|  | |

|  |  |
| --- | --- |
| Protected Member Functions | |
| int | doWork () |
|  | |
| Protected Member Functions inherited from broad.pda.seq.rap.GenomeScoringProgram | |
| String[] | customCommandLineValidation () |
|  | |
| Protected Member Functions inherited from broad.pda.seq.rap.GenomeCommandLineProgram | |
| String[] | customCommandLineValidation () |
|  | |
| void | loadCoordinateSpace () throws IOException |
|  | |
| CoordinateSpace | getCoordinateSpace () |
|  | |

|  |  |
| --- | --- |
| Additional Inherited Members | |
| Protected Attributes inherited from broad.pda.seq.rap.GenomeCommandLineProgram | |
| CoordinateSpace | coordinateSpace |
|  | |

## Member Function Documentation

|  |  |  |  |  |
| --- | --- | --- | --- | --- |
| int broad.pda.seq.rap.SlideAndCalculate.doWork | ( |  | ) |  |

protected

Here is the call graph for this function:

|  |  |  |  |  |
| --- | --- | --- | --- | --- |
| Iterator<? extends WindowScore> broad.pda.seq.rap.SlideAndCalculate.getWindowScoreIterator | ( |  | ) | throws IOException |

Here is the caller graph for this function:

|  |  |  |  |  |  |
| --- | --- | --- | --- | --- | --- |
| static void broad.pda.seq.rap.SlideAndCalculate.main | ( | final String[] | *args* | ) |  |

static

Stock main method.

Parameters
:   |  |  |
    | --- | --- |
    | args | main arguments |

## Member Data Documentation

File broad.pda.seq.rap.SlideAndCalculate.OUTPUT

int broad.pda.seq.rap.SlideAndCalculate.OVERLAP

Double broad.pda.seq.rap.SlideAndCalculate.RPKM\_RATIO\_OFFSET = RatioScore.RPKM\_OFFSET

String broad.pda.seq.rap.SlideAndCalculate.USAGE = "Slides across the genome and counts reads (or calculates ratios for two models). This is currently written for Genomic Space analyses but should be modified to allow other coordinate systems."

int broad.pda.seq.rap.SlideAndCalculate.WINDOW

---

The documentation for this class was generated from the following file:

- java/broad/pda/seq/rap/SlideAndCalculate.java


---

Generated on Fri Aug 15 2014 15:11:03 for ESAT by  

 1.8.7
